# S1P/S1P_2_ Signaling Axis Regulates Both NLRP3 Upregulation and NLRP3 Inflammasome Activation in Macrophages Primed with Lipopolysaccharide

**DOI:** 10.3390/antiox10111706

**Published:** 2021-10-27

**Authors:** Chi-Ho Lee, Ji Woong Choi

**Affiliations:** Laboratory of Neuropharmacology, College of Pharmacy and Gachon Institute of Pharmaceutical Sciences, Incheon 21936, Korea; lch7835@nate.com

**Keywords:** S1P, S1P_2_, LPS, bone marrow-derived macrophage, NLRP3 upregulation, NLRP3 inflammasome activation

## Abstract

The activation of NLRP3 inflammasome is a key factor for various inflammatory diseases. Here, we provide experimental evidence supporting the regulatory role of sphingosine-1-phosphate (S1P) in NLRP3 inflammasome activation in mouse bone-marrow-derived macrophages (BMDMs), along with the S1P receptor subtype involved and underlying regulatory mechanisms. During the priming stage, S1P induced NLRP3 upregulation in BMDMs only when primed with lipopolysaccharide (LPS). In this event, S1P_2_, but not S1P_1_, was involved based on the attenuated NLRP3 upregulation with JTE013 (S1P_2_ antagonist) or S1P_2_ knockdown. During the activation stage, S1P induced NLRP3 inflammasome activation in LPS-primed BMDMs via caspase-1 activation, interleukin 1β maturation, apoptosis-associated speck-like protein containing a CARD (ASC) speck formation, and IL-1β secretion. Such NLRP3 inflammasome activation was blocked by either pharmacological inhibition or genetic knockdown of S1P_2_. NF-κB, PI3K/Akt, and ERK1/2 were identified as effector pathways underlying S1P/S1P_2_ signaling in the regulation of NLRP3 upregulation in LPS-primed BMDMs. Further, reactive oxygen species (ROS) production was dependent on the S1P/S1P_2_ signaling axis in these cells, and the ROS generated regulate NLRP3 inflammasome activation, but not NLRP3 priming. Collectively, our findings suggest that S1P promotes NLRP3 upregulation and NLRP3 inflammasome activation in LPS-primed BMDMs via S1P_2_ and subsequent effector pathways.

## 1. Introduction

NOD-like receptor pyridin domain containing 3 (NLRP3) is an intracellular molecule recognizing various pathogen-associated molecular patterns (PAMPs) and damage-associated molecular patterns (DAMPs) [1,2]. It is an essential component of the NLRP3 inflammasome inducing inflammatory responses [1,2]. When NLRP3 inflammasome is activated, NLRP3 recruits an apoptosis-associated speck-like protein containing a CARD (ASC) and caspase-1, resulting in the maturation and production of proinflammatory cytokines, including interleukin-1β (IL-1β) [1,2]. For NLRP3 inflammasome activation, two signals, known as the priming signal (signal 1) and the activation signal (signal 2), are required, and diverse signaling pathways regulate this activation [1,2]. NLRP3 inflammasome activation has emerged as a pathogenic event in inflammatory, metabolic, neurological, and cardiovascular diseases [3,4].

Sphingosine 1-phosphate (S1P), a bioactive lysophospholipid, regulates various biological activities via specific G protein-coupled receptors (S1P_1_-S1P_5_) [5,6]. S1P is present in cells and tissues and its levels can be increased under disease conditions, including tissue fibrosis, cancer, and cerebral ischemia [7,8,9]. The latter finding indicates that increased levels of S1P may aggravate tissue damage following disease induction via S1P receptors. In the case of NLRP3 inflammasome activation, sphingosine, a precursor of S1P, induces NLRP3 inflammasome activation such as caspase-1 activation and IL-1β secretion in lipopolysaccharide (LPS)-primed peritoneal macrophages [10]. In addition to sphingolipid, lysophosphatidic acid (LPA), another lysophospholipid, induces NLRP3 inflammasome activation in LPS-primed bone marrow-derived macrophages (BMDMs), but not in normal BMDMs [11]. In addition, this finding was notable in psoriasis. in which LPA levels are increased in the lesion sites [11], further supporting the role of bioactive lysophospholipids in facilitating NLRP3 inflammasome activation in activated macrophages. However, it is still unclear whether S1P exerts similar biological effects, as is the role of specific S1P receptors.

In the current study, we found that S1P enhanced NLRP3 upregulation in BMDMs only in the presence of LPS. Further, we found that S1P_2_ contributed to S1P-enhanced NLRP3 upregulation in LPS-primed BMDMs by employing either a pharmacological antagonist (JTE013) or a genetic knockdown with S1P_2_-specific siRNA. It was also found that the S1P/S1P_2_ signaling axis contributed to NLRP3 inflammasome activation in LPS-primed BMDMs because the suppression of S1P_2_ activity attenuated caspase-1 activation, IL-1β maturation, IL-1β secretion, and ASC speck formation. NF-κB, PI3K/Akt, ERK1/2, and reactive oxygen species (ROS) were identified as the key players in the underlying mechanism.

## 2. Materials and Methods

### 2.1. Culture of BMDMs and Treatment

BMDMs were obtained as described previously [11,12]. Briefly, mouse bone marrow cells were obtained from the leg bones of mice (ICR male mice, 8 weeks old; Orient Co. Ltd., Gyeonggi-do, Korea). They were differentiated into BMDMs for 3 days in growth medium (α-MEM (Life Technologies, Carlsbad, CA, USA) supplemented with 10% heat-inactivated fetal bovine serum (FBS, Life Technologies), 1% penicillin/streptomycin (Life Technologies), and 30 ng/mL recombinant mouse macrophage colony-stimulating factor (M-CSF, R&D systems, Minneapolis, MN, USA)) at 37 °C in 5% CO_2_.

To induce NLRP3 inflammasome activation, BMDMs were incubated in an FBS-free growth medium overnight in the presence of the vehicle (0.1% fatty acid free bovine serum albumin, Sigma-Aldrich, St. Louis, MO, USA), primed with LPS (500 ng/mL, Sigma-Aldrich) for 4 h, and exposed to S1P (up to 1 µM, Avanti Polar Lipids, Birmingham, AL, USA) for an additional 1 h.

To antagonize S1P_1_ or S1P_2_, BMDMs were serum-starved overnight in the presence of the vehicle, exposed to W146 (10 µM, Cayman, Ann Arbor, MI, USA) or JTE013 (10 µM, Cayman) for 30 min, and primed with LPS for 4 h. Cells were then exposed to S1P for 1 h. To induce the genetic knockdown of S1P_2_, BMDMs were subjected to transient transfection of S1P_2_ siRNA (Dharmacon, Lafayette, CO, USA) or control siRNA (Dharmacon) with the Lipofectamine^®^ RNAiMAX reagent (Life Technologies) in a growth medium without serum and antibiotics for 6 h. BMDMs were recovered via incubation in a growth medium for 2 days. BMDMs were then serum-starved, primed with LPS, and exposed to S1P. S1P_2_ knockdown by its siRNA was determined by quantitative real-time PCR (qPCR) analysis.

BMDMs were serum-starved overnight and exposed to individual inhibitors of different effector pathways (PI3K, p38, JNK, or ERK1/2) for 30 min to determine the cellular mechanisms. LY294002 (50 µM; Merck Millipore, Burlington, MA, USA), SB203580 (10 µM; Enzo, Farmingdale, NY, USA), SP600125 (10 µM; Sigma-Aldrich), and U0126 (10 µM; Cell Signaling Technology, Danvers, MA, USA) were used to inhibit PI3K, p38, JNK, and ERK1/2, respectively. Cells were also exposed to N-acetylcysteine (NAC, 5 mM; Sigma-Aldrich) for 30 min to inhibit ROS production. Cells were then primed with LPS for 4 h and exposed to S1P for an additional 1 h.

### 2.2. qPCR Analysis

Total RNA was extracted from BMDMs using the RNAiso plus reagent (Takara, Kusatsu, Japan) according to the manufacturer’s instructions. Extracted total RNA (2 µg) was used for cDNA synthesis using a TransScript All-in-One First-Strand cDNA Synthesis SuperMix for qPCR kit (TransGen Biotech, Beijing, China). qPCR analysis was performed using the StepOnePlus^TM^ Real-Time PCR system (Applied Biosystems, Foster city, CA, USA) and the FG Power SYBR Green PCR Master Mix (Life Technologies). GAPDH was used as a house-keeping gene to normalize the values of target genes. Table S1 presents the primer sequences used in this study.

### 2.3. Western Blot Analysis

Protein was extracted from BMDMs using RIPA buffer (ELPIS biotech, Deajeon, Korea) containing a protease inhibitor cocktail and a phosphatase inhibitor. The protein concentration was quantified by the Bradford method using the Bio-Rad protein assay kit (Bio-Rad, Hercules, CA, USA). The protein samples were subjected to SDS-PAGE (10~14% gel) and transferred to the PVDF membrane (Merck Millipore). These membranes were blocked with 5% skim milk (BD Difco, Sparks, MD, USA). They were then incubated with specific primary antibodies against the following molecules: NLRP3 (1:2000, AdipoGen Life Sciences, San Diego, CA, USA), phospho-ERK1/2 (1:1000, Cell Signaling Technology), total ERK1/2 (1:1000, Cell Signaling Technology), phospho-p38 (1:1000, Cell Signaling Technology), total p38 (1:1000, Cell Signaling Technology), phospho-Akt (1:1000, Cell Signaling Technology), total Akt (1:1000, Cell Signaling Technology), IL-1β p17 (mature IL-1β; 1:1000, Cell Signaling Technology), pro-IL-1β (1:1000, Abcam, Cambridge, UK), caspase-1 p20 (cleaved caspase-1; 1:1000, AdipoGen Life Sciences, San Diego, CA, USA), pro-caspase-1 (1:1000, Abcam), and β-actin (1:10,000, Bethyl Laboratories, Montgomery, TX, USA). Membranes were then incubated with appropriate HRP-conjugated secondary antibodies. An enhanced chemiluminescence (ECL) detection kit (Donginbiotech Co., Seoul, Korea) was used to visualize the target protein bands by developing X-ray films manually or using Western blot imaging systems (ImageQuant 800, Amersham, Buckinghamshire, UK). Image J software was used to quantify the expression levels of target proteins.

### 2.4. Immunocytochemistry

BMDMs were fixed with 4% paraformaldehyde for 10 min at 4°C and blocked with 1% FBS containing 0.1% Triton X-100. Cells were incubated with rabbit anti-ASC (1:200, AdipoGen Life Sciences) and mouse anti-NLRP3 (1:100, AdipoGen Life Sciences) primary antibodies overnight at 4 °C followed by incubation with AF488- and Cy3-conjugated secondary antibodies (1:1000, Jackson ImmunoResearch West Grove, PA, USA) for 2 h at room temperature. Cells were stained with DAPI (Carl Roth, Karlsruhe, Germany) and coverslipped with VECTASHIELD^®^ (Vector Laboratories, Burlingame, CA, USA). Images were collected using a confocal microscope (Eclipse A1 Plus, Nikon, Japan) and prepared using Adobe Photoshop Elements 8.

### 2.5. Analysis of NF-κB Translocation

Cytosolic and nuclear protein extracts from BMDMs were prepared using the ProteoExtract^®^ Subcellular Proteome Extraction Kit (Merck) as previously described [12]. Cytosolic and nuclear proteins were separated by SDS-PAGE, transferred to PVDF membranes, blocked with 5% skim milk, and incubated with primary antibodies against NF-κB p65 (1:1000, Cell Signaling Technology), β-actin (1:10,000), and histone H3 (1:1000, Abcam). Membranes were incubated with HRP-conjugated secondary antibodies (1:10,000), and target protein bands were visualized using an ECL detection kit.

### 2.6. ELISA for IL-1β Secretion

The levels of IL-1β protein in cell-free supernatants were determined using IL-1β ELISA kits (Cat#: DY401-05, R&D systems) according to the manufacturer’s instructions.

### 2.7. Determination of Intracellular ROS

Intracellular ROS production was evaluated using a dichlorofluorescein diacetate (DCF-DA) probe (Cat#: D6883, Sigma-Aldrich). Briefly, BMDMs were washed with PBS three times and incubated with DCF-DA (5 μM) for 30 min at 37 °C. The fluorescence of cells was measured using a confocal microscope.

### 2.8. Statistical Analysis

All data analyses were conducted using GraphPad Prism Version 5.02 (GraphPad, La Jolla, CA, USA). Data are expressed as the mean ± S.E.M. Statistical significance was analyzed via Student’s *t*-test between two groups and one-way ANOVA followed by the Newman–Keuls post hoc test for multiple comparisons. Statistical significance was set at a *p*-value less than 0.05.

## 3. Results

### 3.1. S1P Enhances NLRP3 Upregulation in LPS-Primed Macrophages

**Figure 1:**
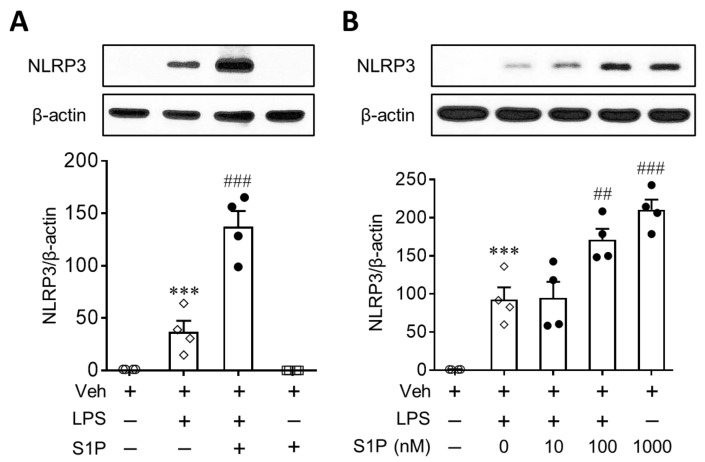
S1P enhances NLRP3 upregulation in LPS-primed BMDMs. Cells were treated with LPS (500 ng/mL) for 4 h and then exposed to S1P (1 μM) for an additional 1 h. In some cases, cells were exposed to S1P for 1 h without priming with LPS. (**A**) Effects of S1P on NLRP3 expression in BMDMs in the presence or absence of LPS were analyzed by Western blot. (**B**) Concentration-dependent effects of S1P (0.01, 0.1, and 1 μM) on NLRP3 expression in LPS-primed BMDMs were analyzed by Western blot. *n* = 4 per group. *** *p* < 0.001 versus control BMDMs (Veh). ## *p* < 0.01, and ### *p* < 0.001 versus LPS-primed BMDMs.

We investigated whether S1P influenced the expression of NLRP3 in LPS-primed BMDMs. Cells were treated with LPS (500 ng/mL, 4 h) and then exposed to S1P (1 µM) for an additional 1 h. When LPS-primed cells were exposed to S1P, the expression of the NLRP3 protein was markedly upregulated by approximately 3-fold compared to cells treated with LPS only (Figure 1A). However, S1P alone did not induce NLRP3 upregulation in normal BMDMs (Figure 1A). Even when cells were exposed to S1P for a longer duration (24 h, 1 µM) or at higher concentration (5 µM, 24 h), S1P itself did not induce NLRP3 upregulation in normal BMDMs (Figure S1). S1P-mediated NLRP3 upregulation in LPS-primed BMDMs was concentration dependent: 0.1 or 1 µM S1P induced a significant upregulation of NLRP3 compared to cells treated with LPS alone (Figure 1B). These data clearly demonstrated that S1P enhanced NLRP3 upregulation in LPS-primed macrophages.

### 3.2. S1P_2_ Is Required for S1P-Enhanced NLRP3 Upregulation in LPS-Primed Macrophages

**Figure 2:**
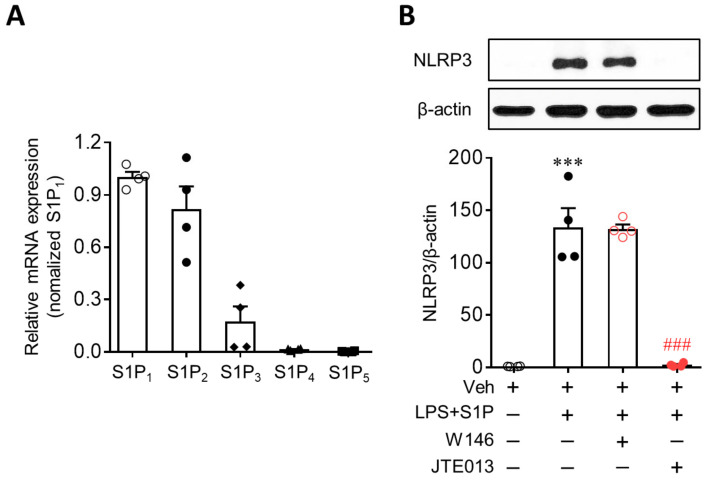
JTE013, an S1P_2_ antagonist, attenuates S1P-enhanced NLRP3 upregulation in LPS-primed BMDMs. (**A**) Expression of S1P receptors at mRNA levels was determined in BMDMs using qRT-PCR analysis. *n* = 4 per group. (**B**) Effects of W146 (an S1P_1_ antagonist, 1 μM) or JTE013 (an S1P_2_ antagonist, 10 μM) on NLRP3 expression were analyzed by Western blot. Cells were pretreated with W146 or JTE013 for 30 min. Cells were then primed with LPS (500 ng/mL) for 4 h and exposed to S1P (1 μM) for 1 h. *n* = 4 per group. *** *p* < 0.001 versus control BMDMs (Veh). ### *p* < 0.001 versus LPS/S1P-treated BMDMs (LPS + S1P).

**Figure 3:**
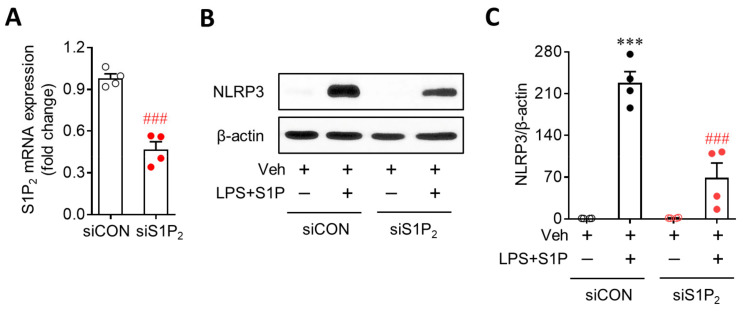
S1P_2_ knockdown attenuates S1P-induced NLRP3 upregulation in LPS-primed BMDMs. BMDMs transfected with non-target control siRNA (siCON) or S1P_2_-specific siRNA (siS1P_2_) were primed with LPS (500 ng/mL) for 4 h and exposed to S1P (1 μM) for 1 h. (**A**) S1P_2_ knockdown efficiency by its specific siRNA was determined by qRT-PCR analysis. *n* = 4 per group. ### *p* < 0.001 versus control siRNA (siCON)-transfected BMDMs. (**B**,**C**) Effects of S1P_2_ knockdown on NLRP3 upregulation were analyzed by Western blot. Representative blots of NLRP3 (**B**) and quantification (**C**). *n* = 4 per group. *** *p* < 0.001 versus control BMDMs transfected with control siRNA (siCON + Veh). ### *p* < 0.001 versus LPS/S1P-treated BMDMs transfected with control siRNA (siCON + LPS + S1P).

Next, we investigated whether S1P-enhanced NLRP3 upregulation in LPS-primed BMDMs was mediated by S1P receptors. In BMDMs, two of the S1P receptors, S1P_1_ and S1P_2_, were highly expressed, as evidenced by the mRNA expression levels (Figure 2A). S1P_3_ and S1P_4_ were marginally expressed, whereas S1P_5_ was hardly detected (Figure 2A). To determine which of the two S1P receptors mediated the S1P-enhanced NLRP3 upregulation in LPS-primed BMDMs, cells were pretreated with a specific antagonist for S1P_1_ (W146, 10 µM) or S1P_2_ (JTE013, 10 µM) for 30 min prior to LPS exposure. Interestingly, inhibiting S1P_2_, but not S1P_1_, markedly attenuated S1P-enhanced NLRP3 upregulation in LPS-primed BMDMs (Figure 2B). This regulatory role of S1P_2_ was further confirmed using cells with genetically suppressed S1P_2_ expression. S1P_2_ knockdown via transfection with specific siRNA (Figure 3A) significantly attenuated S1P-enhanced NLRP3 upregulation in LPS-primed BMDMs (Figure 3B,C). These independent data derived from pharmacological or genetic approaches clearly demonstrate that the S1P/S1P_2_ signaling axis represents a novel regulator of NLRP3 upregulation in LPS-primed macrophages.

### 3.3. S1P/S1P_2_ Signaling Axis Activates NLRP3 Inflammasome in LPS-Primed Macrophages

**Figure 4:**
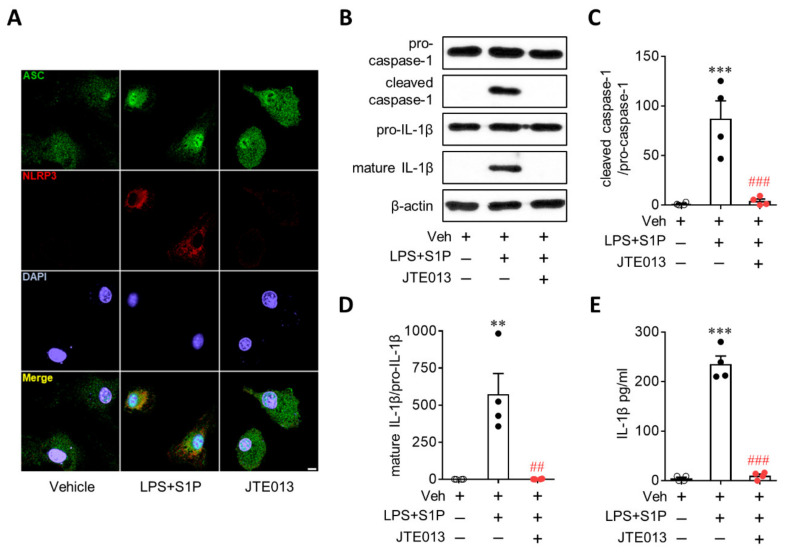
JTE013 treatment attenuates S1P-induced NLRP3 inflammasome activation in LPS-primed BMDMs. Cells were pretreated with JTE013 (10 μM) for 30 min. Cells were then primed with LPS (500 ng/mL) for 4 h and exposed to S1P (1 μM) for 1 h. (**A**) Effects of JTE013 on ASC speck formation were determined by double immunofluorescence for ASC (green) and NLRP3 (red) in LPS/S1P-treated BMDMs (LPS+S1P). Representative images of ASC speck formation in NLRP3-positive BMDMs. Scale bar, 10 μm. (**B**–**D**) Effects of JTE013 on caspase-1 activation and IL-1β maturation were analyzed by Western blot. (**B**) Representative blots of pro-caspase-1, cleaved caspase-1, pro-IL-1β, and mature IL-1β. Quantification of caspase-1 activation (**C**) and IL-1β maturation (**D**). (**E**) Effects of JTE013 on IL-1β secretion were measured via ELISA. *n* = 4 per group. ** *p* < 0.01 and *** *p* < 0.001 versus control BMDMs (Veh). ## *p* < 0.01 and ### *p* < 0.001 versus LPS/S1P-treated BMDMs (LPS + S1P).

**Figure 5:**
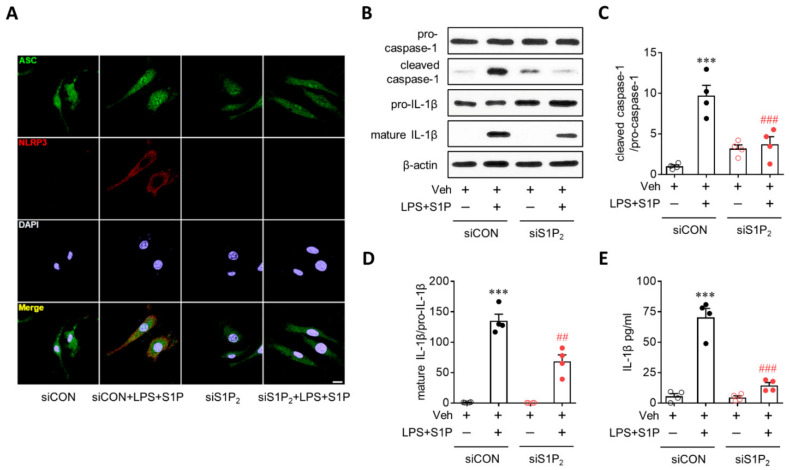
S1P_2_ knockdown attenuates S1P-induced NLRP3 inflammasome activation in LPS-primed BMDMs. BMDMs transfected with non-target control siRNA (siCON) or S1P_2_-specific siRNA (siS1P_2_) were primed with LPS (500 ng/mL) for 4 h and exposed to S1P (1 μM) for 1 h. (**A**) Effects of S1P_2_ knockdown on ASC speck formation were determined by double immunofluorescence for ASC (green) and NLRP3 (red) in LPS/S1P-treated BMDMs (LPS+S1P). Representative images of ASC speck formation in NLRP3-positive BMDMs. Scale bar, 10 μm. (**B**–**D**) Effects of S1P_2_ knockdown on caspase-1 activation and IL-1β maturation were analyzed by Western blot. (**B**) Representative blots of pro-caspase-1, cleaved caspase-1, pro-IL-1β, and mature IL-1β. Quantification of caspase-1 activation (**C**) and IL-1β maturation (**D**). (**E**) Effects of S1P_2_ knockdown on IL-1β secretion were measured by ELISA. *n* = 4 per group. *** *p* < 0.001 versus control BMDMs transfected with control siRNA (siCON + Veh). ## *p* < 0.01 and ### *p* < 0.001 versus LPS/S1P-treated BMDMs transfected with control siRNA (siCON + LPS + S1P).

NLRP3 upregulation contributes to the activation of the NLRP3 inflammasome as a priming signal, leading to IL-1β production. During NLRP3 inflammasome activation, an activation signal is required in addition to a priming signal. Therefore, it is possible that the S1P/S1P_2_ signaling axis also activates the NLRP3 inflammasome beyond its role as a priming signal to induce NLRP3 upregulation. To address this possibility, we first determined NLRP3-dependent ASC recruitment, which is a critical step in NLRP3 inflammasome activation [1,2]. S1P exposure induced the formation of ASC speckles in LPS-primed BMDMs (Figure 4A), especially in cells where NLRP3 was upregulated (Figure 4A). This ASC speck formation in LPS/S1P-treated BMDMs was attenuated by suppressing S1P_2_ activity with the JTE013 treatment (Figure 4A). We further determined the activation of two additional molecules playing a key role in the activation of the NLRP3 inflammasome, that is caspase-1 activation and subsequent IL-1β maturation, in LPS-induced BMDMs. S1P exposure markedly upregulated cleaved caspase-1 (Figure 4B,C) and mature IL-1β p17 in LPS-primed BMDMs (Figure 4B,D) without affecting the expression of pro-caspase-1 or pro-IL-1β (Figure 4B). This marked elevation was completely attenuated by suppressing S1P_2_ activity: JTE013 treatment attenuated S1P-induced caspase-1 activation (Figure 4B,C) and IL-1β maturation (Figure 4B,D) in LPS-primed BMDMs. In addition, S1P induced IL-1β secretion from LPS-primed BMDMs (Figure 4E), which was also markedly attenuated by the JTE013 treatment (Figure 4E). These critical roles of S1P_2_ in NLRP3 inflammasome activation were validated by genetic knockdown using S1P_2_ siRNA. S1P_2_ knockdown significantly attenuated S1P-triggered ASC speck formation (Figure 5A), caspase-1 activation (Figure 5B,C), IL-1β maturation (Figure 5B,D), and IL-1β secretion (Figure 5E) in LPS-primed BMDMs. These data from pharmacological and genetic approaches clearly demonstrated the critical role of the S1P/S1P_2_ signaling axis in triggering NLRP3 inflammasome activation in LPS-primed macrophages.

### 3.4. NF-κB Activation Mediates S1P/S1P_2_ Signaling-Directed NLRP3 Upregulation in LPS-Primed Macrophages

**Figure 6:**
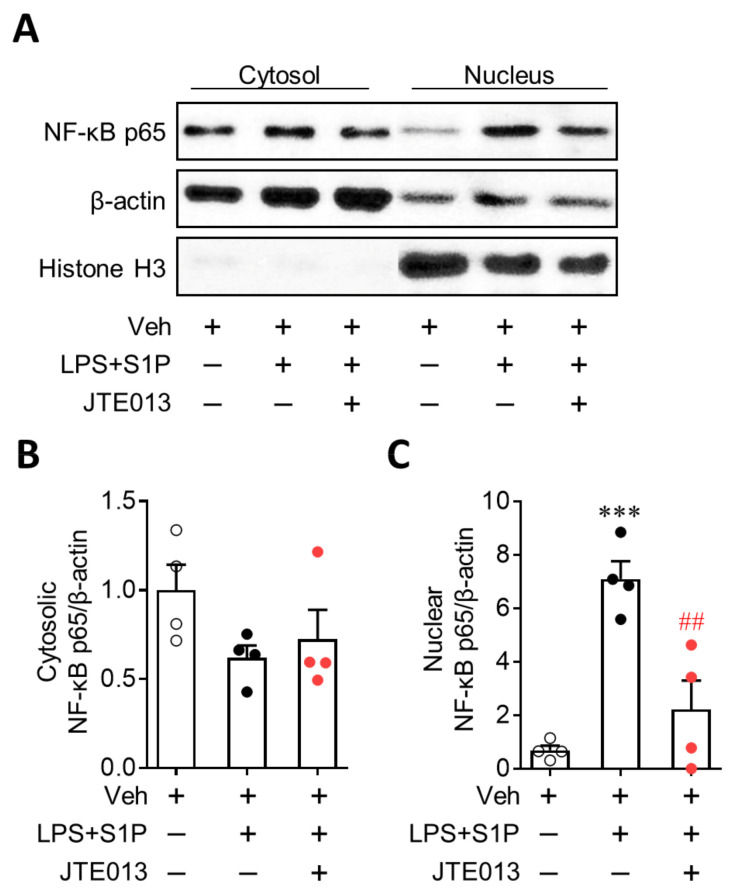
S1P_2_ regulates NF-κB activation in LPS-primed BMDMs followed by S1P exposure. Cells were pretreated with JTE013 (10 μM) for 30 min. Cells were then primed with LPS (500 ng/mL) for 4 h and exposed to S1P (1 μM) for 1 h. Effects of JTE013 on NF-κB translocation from the cytosol to the nucleus were analyzed by Western blot. Representative blots of cytosolic and nuclear NF-κB p65 (**A**) and quantification (**B**, cytosolic NF-κB p65; **C**, nuclear NF-κB p65) are shown. *n* = 4 per group. *** *p* < 0.001 versus control BMDMs (Veh). ## *p* < 0.01 versus LPS/S1P-treated BMDMs (LPS + S1P).

NF-κB plays a critical role in NLRP3 upregulation, acting as a priming signal [13,14]. Therefore, we investigated the role of S1P_2_ in NF-κB activation by determining the nuclear translocation of NF-κB. S1P exposure robustly enhanced the expression of NF-κB (p65) in the nucleus without affecting its cytosolic expression in LPS-primed BMDMs (Figure 6), demonstrating enhanced nuclear translocation of NF-κB by S1P in LPS-primed cells. The suppression of S1P_2_ activity by JTE013 significantly attenuated this enhanced translocation (Figure 6). These results demonstrated that S1P/S1P_2_ signaling upregulates NLRP3 expression by activating a representative priming signal, NF-κB, in LPS-primed macrophages.

### 3.5. Activation of PI3K and ERK1/2 Mediates S1P/S1P_2_ Signaling-Directed NLRP3 Upregulation in LPS-Primed Macrophages

**Figure 7:**
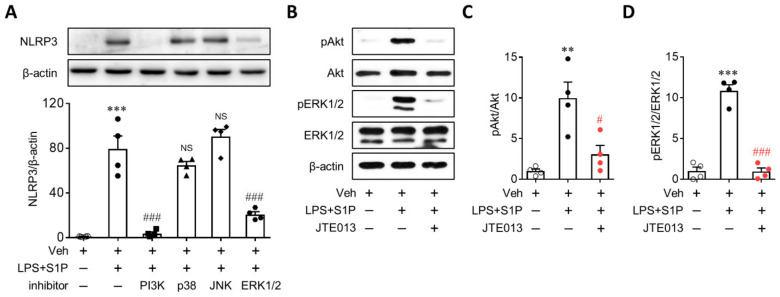
S1P_2_ regulates activation of PI3K and ERK1/2 in LPS-primed BMDMs followed by S1P exposure. Cells were primed with LPS (500 ng/mL) for 4 h and exposed to S1P (1 μM) for 1 h. (**A**) Effects of an inhibitor of PI3K/Akt, p38, JNK, or ERK1/2 on NLRP3 upregulation were analyzed by Western blot. Cells were pretreated with LY294002 (50 μM, a PI3K/Akt inhibitor), p38 SB203580 (10 μM, a p38 MAPK inhibitor), SP600125 (10 μM, a JNK inhibitor), or U0126 (10 μM, an ERK1/2 inhibitor) for 30 min prior to LPS priming. Representative blots and quantification data are shown. (**B**–**D**) Effects of JTE013 on activation of PI3K/Akt and ERK1/2 were analyzed by Western blot. Cells were treated with JTE013 (10 μM) for 30 min, primed with LPS, and exposed to S1P. Representative blots of phosphorylated Akt (pAkt), total Akt (Akt), phosphorylated ERK1/2 (pEKR1/2), and total ERK1/2 (ERK1/2) (**B**) and quantification (**C**, PI3K/Akt activation; **D**, ERK1/2 activation). *n* = 4 per group. ** *p* < 0.01 and *** *p* < 0.001 versus control BMDMs (Veh). # *p* < 0.05 and ### *p* < 0.001 versus LPS/S1P-treated BMDMs (LPS + S1P).

S1P_2_ influenced PI3K and MAPKs effector pathways [15], all of which mediated NLRP3 upregulation [16,17,18,19,20]. To determine the role of downstream signaling pathways after S1P_2_ activation in NLRP3 upregulation, BMDMs were pretreated with various inhibitors of PI3K (LY294002, 50 µM), p38 (SB203580, 10 µM), JNK (SP600125, 10 µM), and ERK1/2 (U0126, 10 µM) for 30 min prior to LPS priming. Treatment with either PI3K or the ERK1/2 inhibitor significantly attenuated S1P-enhanced NLRP3 upregulation in LPS-primed BMDMs (Figure 7A), whereas other inhibitors of p38 or JNK did not (Figure 7A). Next, we established that the S1P/S1P_2_ signaling axis affected the activation of PI3K and ERK1/2. S1P exposure induced marked phosphorylation of Akt (Figure 7B,C) and ERK1/2 (Figure 7B,D) in LPS-primed BMDMs, both of which were significantly attenuated via the suppression of S1P_2_ activity with JTE013 (Figure 7B–D). These results demonstrated that the S1P/S1P_2_ signaling axis directed NLRP3 upregulation by activating PI3K and ERK1/2 in LPS-primed macrophages.

### 3.6. ROS Mediate S1P/S1P_2_ Signaling-Directed NLRP3 Inflammasome Activation in LPS-Primed Macrophages

**Figure 8:**
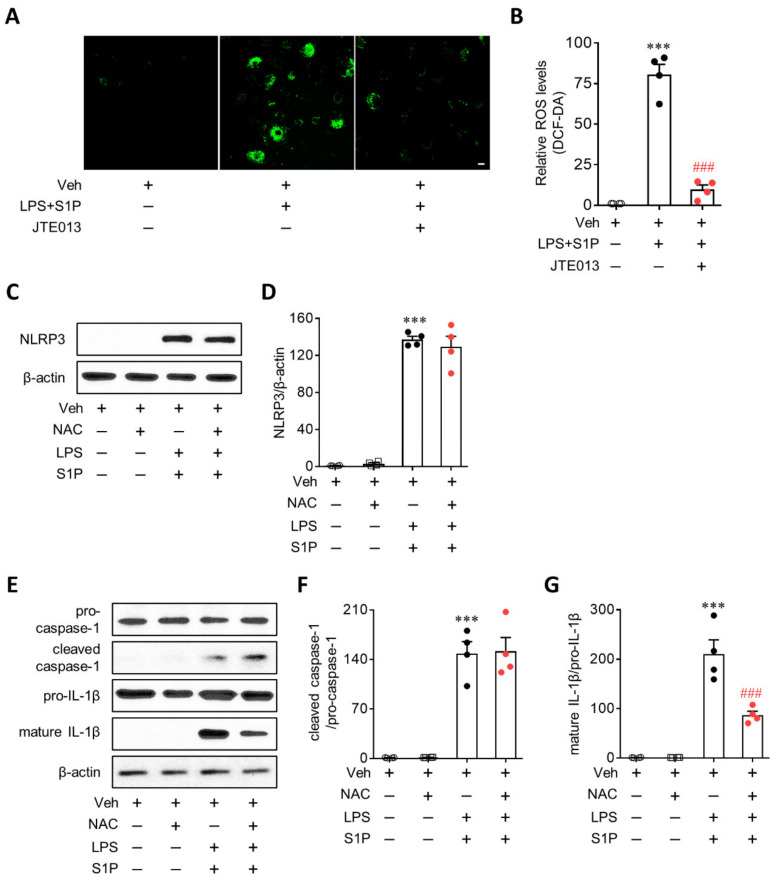
S1P_2_ regulates intracellular ROS production from LPS-primed BMDMs followed by S1P exposure. Cells were treated with JTE013 (10 μM) or NAC (5 mM) for 30 min. BMDMs were then primed with LPS (500 ng/mL) for 4 h and exposed to S1P (1 μM) for 1 h. (**A**,**B**) Effects of JTE013 on intracellular ROS production were analyzed by DCF-DA staining of LPS/S1P-treated BMDMs (LPS + S1P). Representative images of cells bearing DCF (**A**) and quantification of ROS levels (**B**). Scale bar, 10 μm. *n* = 4 per group. *** *p* < 0.001 versus control BMDMs (Veh). ### *p* < 0.001 versus LPS/S1P-treated BMDMs (LPS+S1P). (**C**,**D**) Effects of NAC on NLRP3 upregulation were determined by Western blot in LPS/S1P-treated BMDMs (LPS+S1P). Representative blot of NLRP3 (**C**) and quantification (**D**). *n* = 4 per group. *** *p* < 0.001 versus control BMDMs (Veh). (**E**–**G**) Effects of NAC on NLRP3 inflammasome activation were analyzed by Western blot in LPS/S1P-treated BMDMs (LPS+S1P). Representative blots of pro-caspase-1, cleaved caspase-1, pro-IL-1β, and mature IL-1β € and quantification (**F**, caspase-1 activation; **G**, IL-1β maturation). *n* = 4 per group. *** *p* < 0.001 versus control BMDMs (Veh). ### *p* < 0.001 versus LPS/S1P-treated BMDMs (LPS + S1P).

ROS contribute to both NLRP3 priming and NLRP3 inflammasome activation [2,21]. Therefore, whether S1P_2_ influences ROS production in LPS/S1P-stimulated BMDMs was determined using DCF-DA. S1P exposure induced a robust production of ROS from LPS-primed BMDMs (Figure 8A,B). The suppression of S1P_2_ activity by JTE013 significantly attenuated ROS production (Figure 8A,B). Next, the role of ROS in NLRP3 priming and/or NLRP3 inflammasome activation was investigated using a ROS inhibitor, NAC. Pretreatment with NAC (5 mM; 30 min prior to LPS priming) did not influence S1P-enhanced NLRP3 upregulation in LPS-primed BMDMs (Figure 8C,D), suggesting that ROS are not involved in the NLRP3 priming step. However, NAC treatment significantly attenuated S1P-triggered NLRP3 inflammasome activation (Figure 8E–G). Interestingly, such attenuation was attributed to the effect on IL-1β maturation, but not on caspase-1 activation, because NAC treatment reduced the expression of mature IL-1β (Figure 8E,G) without altering the levels of cleaved caspase-1 (Figure 8E,F) in LPS/S1P-stimulated BMDMs. These results demonstrated the role of ROS in S1P/S1P_2_ signaling axis-regulated NLRP3 inflammasome activation in LPS-primed macrophages.

## 4. Discussion

**Figure 9:**
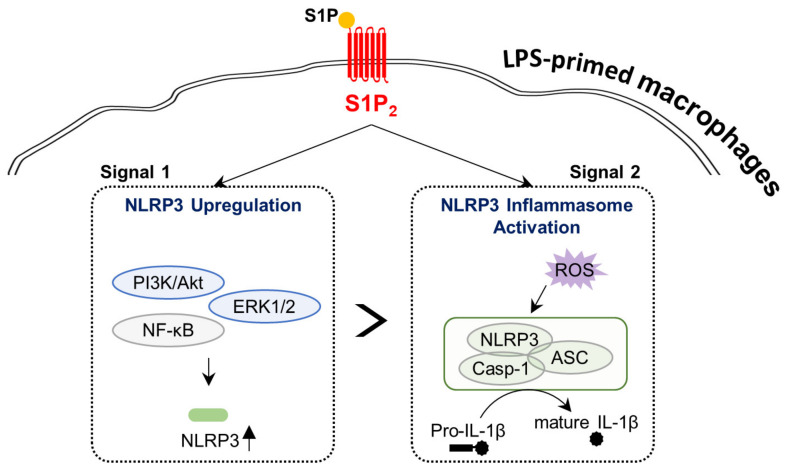
Schematic diagram describing the regulation of the activation of NLRP3 inflammasome by the S1P/S1P_2_ signaling axis in BMDMs. S1P contributes to both NLRP3 upregulation (Signal 1) and NLRP3 inflammasome activation (Signal 2; ASC speck formation, caspase-1 activation, IL-1β maturation, and IL-1β secretion) in BMDMs only when cells were primed with LPS. S1P_2_ plays a pivotal role in such S1P-driven NLRP3 upregulation and NLRP3 inflammasome activation in LPS-primed BMDMs. The S1P/S1P_2_ signaling axis regulates the activation of NF-κB, PI3K/Akt, and ERK1/2 in NLRP3 upregulation. In addition, the S1P/S1P_2_ signaling axis regulates ROS production in NLRP3 inflammasome activation.

NLRP3 inflammasome activity is a crucial event in the pathogenesis of various inflammatory diseases [1,2,3,4]. It can be sequentially activated by signal 1 (a priming signal) and signal 2 (an activation signal), which regulate NLRP3 upregulation and NLRP3 inflammasome activation, such as complex assembly, caspase-1 activation, and IL-1β maturation, respectively [1,2]. The current study indicated that S1P signaling regulated both events (NLRP3 upregulation and NLRP3 inflammasome activation) in macrophages based on in vitro studies using cultured BMDMs (Figure 9). Exogenous S1P exposure affected NLRP3 priming only when macrophages were primed with LPS, but not when they were unprimed. S1P_2_ contributed to this S1P-driven NLRP3 upregulation in LPS-primed macrophages. In addition to NLRP3 priming, exogenous S1P exposure induced NLRP3 inflammasome activation in LPS-primed macrophages as evidenced by ASC speck formation, caspase-1 activation, IL-1β maturation, and IL-1β secretion. This S1P-driven NLRP3 inflammasome activation in LPS-primed macrophages was regulated by S1P_2_. NF-κB, ERK1/2, and PI3K-mediated S1P/S1P_2_ signaling axis-regulated NLRP3 upregulation and ROS production played a role in S1P/S1P_2_ signaling axis-regulated NLRP3 inflammasome activation.

Notably, the current study demonstrated the regulatory role of S1P in NLRP3 priming in macrophages only in primed cells. In fact, S1P itself did not induce NLRP3 upregulation in mouse BMDMs under the experimental conditions (1 µM S1P exposure for 1 h) of the current study. Further, neither longer S1P exposure (24 h) or a higher concentration of S1P (5 µM) induced NLRP3 upregulation in unprimed BMDMs. Instead, S1P exposure enhanced NLRP3 upregulation in LPS-primed BMDMs. Therefore, findings from the current study indicate that S1P potentiates NLRP3 upregulation only in primed macrophages, as also reported by an independent group, albeit partially [22]. Inhibition of sphingosine kinases, enzymes for S1P synthesis, with SKII abrogated the NLRP3 upregulation at the transcription level in primary human macrophages [22], suggesting that endogenously produced S1P was involved in NLRP3 priming. In contrast, another group reported that S1P itself induced NLRP3 upregulation in bone-marrow monocytes/macrophages (BMMs) [23,24]. S1P exposure (1 µM) for 4 h was enough to induce NLRP3 upregulation based on the contrasting findings [23]. NLRP3 was upregulated by S1P itself in BMMs, which were differentiated from mouse bone-marrow cells cultured with the L929-conditioned medium [23,24], but not in BMDMs differentiated from the same bone-marrow cells with recombinant M-CSF. Although the L929-conditioned medium contains large amounts of M-CSF, it can also contain other molecules that may affect cellular function. In fact, L929-conditioned medium-differentiated macrophages released larger amounts of pro-inflammatory cytokines in response to LPS than M-CSF-differentiated macrophages [25].

In addition to NLRP3 priming, S1P activates the NLRP3 inflammasome in LPS-primed BMDMs. It induced ASC speck formation, caspase-1 activation, and IL-1β maturation in primed cells without affecting the expression levels of pro-caspase-1 and pro-IL-1β. In unprimed BMMs, S1P itself activated the NLRP3 inflammasome differently by inducing the upregulation of both the precursor and activated forms of caspase-1 and IL-1β [23,24]. In LPS-primed peritoneal macrophages, sphingosine, a precursor of S1P, can induce NLRP3 inflammasome activation such as IL-1β secretion [26]. Similarly, in LPS-primed primary human macrophages or mouse BMDMs that were differentiated with M-CSF and GM-CSF, the blockade of S1P production with SKII abrogated IL-1β secretion by aluminum hydroxide (AlOH), which is a known trigger of the NLRP3 inflammasome assembly [22]. However, these two independent groups [10,22] reported contrasting results involving S1P. In LPS-primed peritoneal macrophages, S1P induced IL-1β secretion only at a very high concentration (40 µM) [10]. In LPS-primed human macrophages, 1 µM of S1P did not induce IL-1β secretion [22]. Although the current study demonstrated that S1P activated the NLRP3 inflammasome in primed BMDMs, it may be noteworthy that S1P regulated NLRP3 inflammasome activation differently depending on the type of macrophages used experimentally.

The receptor-mediated S1P signaling regulates NLRP3 inflammasome activation [23,24,27,28]. The current expression profiling of S1P receptors in BMDMs demonstrated that S1P_1_ and S1P_2_ were abundant. Importantly, the current study identified S1P_2_ as the S1P receptor subtype contributing to S1P-enhanced NLRP3 priming in LPS-primed BMDMs using an antagonist (JTE013) and a specific siRNA. In addition to the priming event, the current study demonstrated that S1P_2_ was responsible for S1P-driven NLRP3 inflammasome activation in LPS-primed BMDMs. The currently identified role of S1P_2_ in the regulation of NLRP3 activity is supported by recent studies [23,24]. Suppressing S1P_2_ activity with JTE013 treatment attenuated NLRP3 priming and NLPR3 inflammasome activation in S1P-treated BMMs in vitro and bile duct ligation-induced cholestatic liver injury in vivo [23,24]. In addition to S1P_2_, other receptor subtypes regulate NLRP3 activity. However, in the current study, S1P_1_, which is another highly expressed receptor subtype in BMDMs, was not associated with NLRP3 priming because its antagonist (W146) did not attenuate S1P-enhanced NLRP3 upregulation in LPS-primed BMDMs. Similarly, S1P_1_ was highly expressed on BMMs, but it did not affect S1P-induced NLRP3 priming and NLPR3 inflammasome activation [24]. However, the role of S1P_1_ in NLRP3 inflammasome activity is disrupted in certain cases [27,28] compared with previous [24] and current findings. In fact, genetic deletion of S1P_1_ in macrophages prevented pulmonary metastasis and lymphangiogenesis via NLRP3 upregulation and IL-1β production [28]. W146 also suppressed both events in BMDMs exposed to LPS/AlOH [23]. The impact of S1P_1_ on NLRP3 inflammasome activation was also reported in spinal cord injury. Intrathecal injection of SEW2871, a selective agonist of S1P_1_, resulted in mechanoallodynia in the dorsal horn of the spinal cord via NLRP3 upregulation and NLRP3 inflammasome activation [27]. In the case of S1P_3_, a previous study demonstrated that it was also highly expressed on BMMs but was not involved in S1P-induced NLRP3 priming and NLPR3 inflammasome activation [24]. Although the role of other S1P receptor subtypes (i.e., S1P_4_ and S1P_5_) in NLRP3 activity has yet to be investigated, it remains possible that such receptors participate in NLRP3 priming and activation of its inflammasome.

Various signaling molecules influence NLRP3 priming [13,14,16,17,18,19,20]. NF-κB activation is an especially critical event in priming [13,14]. In the current study, JTE013 treatment attenuated NF-κB activation in LPS/S1P-treated BMDMs, indicating that it was the underlying mechanism in S1P/S1P_2_ signaling-dependent NLRP3 priming in these cells. In addition to NF-κB, PI3K and MAPK (ERK1/2, p38, and JNK) pathways can influence NLRP3 priming [16,17,18,19,20], and they are also downstream pathways of S1P_2_ [15]. The current study showed that PI3K and ERK1/2, but not p38 and JNK, mediated the S1P/S1P_2_ signaling-dependent NLRP3 upregulation in LPS-primed BMDMs. When compared to a previous study [23], it may be either similar or reversed. In BMMs, ERK1/2 and JNK were involved in NLRP3 upregulation and p38 was involved in pro-IL-1β upregulation, indicating that all three MAPKs influence S1P-induced NLRP3 priming [23]. However, in the current study, ERK1/2 was the only MAPK mediating NLRP3 upregulation in LPS/S1P-treated BMDMs. Further, PI3K was critical for NLRP3 upregulation in LPS/S1P-treated BMDMs based on the current study, but not in S1P-treated BMMs [23]. Although there are discrepancies in the signaling molecules involved, it is clear that both ERK1/2 and PI3K contribute to S1P/S1P_2_ signaling-dependent NLRP3 upregulation in LPS-primed BMDMs.

It is well-known that ROS serve as signal 2 in NLRP3 inflammasome activation by triggering the NLRP3 inflammasome assembly [2,29,30]. ROS also serve as signal 1 to induce NLRP3 priming [21,31,32,33]. In addition to such a regulatory role in NLRP3 priming and activation of its inflammasome, ROS mediate the biological function of S1P_2_. Indeed, suppressing S1P_2_ activity with either JTE013 or S1P_2_-specific siRNA attenuates ROS production [34,35,36], suggesting that S1P_2_ triggers ROS generation. In the current study, JTE013 treatment attenuated ROS production from LPS/S1P-treated BMDMs, indicating that ROS production represents an underlying mechanism for NLRP3 priming and/or NLRP3 inflammasome activation in these cells. Results from the current study using NAC indicated that ROS participated solely in NLRP3 inflammasome activation of LPS/S1P-treated BMDMs via IL-1β maturation.

## 5. Conclusions

In summary, the current study demonstrated that S1P enhanced both NLRP3 priming and NLRP3 inflammasome activation in LPS-stimulated macrophages. Further, S1P_2_ played a pivotal role in the regulation of such events via activating pathways of NF-κB, PI3K, ERK1/2, and ROS. Based on the established roles of the NLRP3 inflammasome and S1P_2_ demonstrated in independent studies involving several disease types, such as liver fibrosis [37,38], cerebral ischemia [39,40], and psoriasis [41,42], the current findings suggest a possible clue for the pathogenic mechanism underlying the role of the S1P/S1P_2_ signaling axis in tissue injuries. It might also be interesting to pursue the role of this signaling axis in the regulation of macrophage polarization, which is a critical event for immune responses under various tissue injuries since NLRP3 is associated with M1 polarization of macrophages [43,44,45].

## Data Availability

Data is contained within the article or Supplementary Materials.

## Supplementary Materials

The following are available online at https://www.mdpi.com/article/10.3390/antiox10111706/s1, Figure S1: S1P itself does not affect NLRP3 upregulation in normal BMDMs. Cells were exposed to LPS (500 ng/mL, a positive control) or S1P (1 and 5 μM) for 24 h. Effects of S1P on NLRP3 expression in BMDMs were determined by Western blot analysis. *n* = 4 per group. *** *p* < 0.001 versus control BMDMs (Veh). Table S1: Primer sets used for qPCR analysis in this study.
