# Supplementary material for: S1P/S1P2 Signaling Axis Regulates Both NLRP3 Upregulation and NLRP3 Inflammasome Activation in Macrophages Primed with Lipopolysaccharide
Source: Antioxidants (Basel). 2021 Oct 27;10(11):1706. doi: 10.3390/antiox10111706 (PMC8614891; doi:10.3390/antiox10111706)
Supplement: Supplementary file 1 [file antioxidants-10-01706-s001.zip › antioxidants-1425679-supplementary.pdf]

antioxidants

MDPI

Supplementary Material

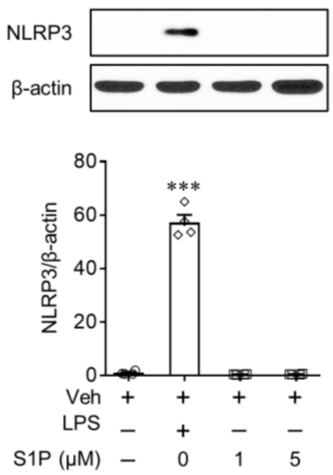

Figure S1. S1P itself does not affect NLRP3 upregulation in normal BMDMs. Cells were exposed to LPS (500 ng/mL, a positive control) or S1P (1 and 5 μM) for 24 h. Effects of S1P on NLRP3 expression in BMDMs were determined by Western blot analysis. $n = 4$ per group. *** $p < 0.001$ versus control BMDMs (Veh).

Table S1. Primer sets used for qPCR analysis in this study.

Gene.	Accession Number	Sequence
$S1P_1$ (M)	NM_007901.5	Forward: 5′-AGG GAA CTT TGC GAG TGA G-3′<br>Reverse: 5′-GTT ACA GCA AAG CCA GGT CAG-3′
$S1P_2$ (M)	NM_010333.4	Forward: 5′-ATA GAC CGA GCA CAG CCA AC-3′<br>Reverse: 5′-GTG TTC CAG AAC CTT CTC AGG-3′
$S1P_3$ (M)	NM_010101.4	Forward: 5′-TTG CAG AAC GAG AGC CTA TT-3′<br>Reverse: 5′-TTC CCG GAG AGT GTC ATT TC-3′
$S1P_4$ (M)	NM_010102.2	Forward: 5′-ACC TTC AGT CTG CTC TTC ACG-3′<br>Reverse: 5′-AAG AGC ACA TAG CCC TTG GAG-3′
$S1P_5$ (M)	NM_053190.2	Forward: 5′-AGA TTT CCA ATA GCC GCT CTC-3′<br>Reverse: 5′-AGC TTG CCG GTG TAG TTG TAG-3′
β-actin (M)	NM_007393.5	Forward: 5′-AGC CTT CCT TCT TGG GTA TG-3′<br>Reverse: 5′-CTT CTG CAT CCT GTC AGC AA-3′
GAPDH (M)	NM_001289726.1	Forward: 5′-TTG ATG GCA ACA ATC TCC AC-3′<br>Reverse: 5′-CGT CCC GTA GAC AAA ATG GT-3′
